# Ecological speciation in postglacial European whitefish: rapid adaptive radiations into the littoral, pelagic, and profundal lake habitats

**DOI:** 10.1002/ece3.867

**Published:** 2013-11-11

**Authors:** Kim Præbel, Rune Knudsen, Anna Siwertsson, Markku Karhunen, Kimmo K Kahilainen, Otso Ovaskainen, Kjartan Østbye, Stefano Peruzzi, Svein-Erik Fevolden, Per-Arne Amundsen

**Affiliations:** 1 Department of Arctic and Marine Biology, University of TromsøTromsø, N-9037, Norway; 2 Department of Biosciences, University of HelsinkiPO Box 65, Helsinki, FI-00014, Finland; 3 Department of Environmental Sciences, University of HelsinkiPO Box 65, Helsinki, FI-00014, Finland; 4 Kilpisjärvi Biological Station, University of HelsinkiKäsivarrentie 14622, Kilpisjärvi, FI-99490, Finland; 5 Department of Biology, Centre for Ecological and Evolutionary Synthesis (CEES), University of OsloPO Box 1066, Blindern, Oslo, N-0315, Norway; 6 Department of Forestry and Wildlife Management, Hedmark University CollegeCampus Evenstad, Elverum, NO-2418, Norway

**Keywords:** *Coregonus lavaretus*, gill raker, phenotype–genotype correlation, postglacial fish, reproductive isolation

## Abstract

Understanding how a monophyletic lineage of a species diverges into several adaptive forms has received increased attention in recent years, but the underlying mechanisms in this process are still under debate. Postglacial fishes are excellent model organisms for exploring this process, especially the initial stages of ecological speciation, as postglacial lakes represent replicated discrete environments with variation in available niches. Here, we combine data of niche utilization, trophic morphology, and 17 microsatellite loci to investigate the diversification process of three sympatric European whitefish morphs from three northern Fennoscandian lakes. The morphological divergence in the gill raker number among the whitefish morphs was related to the utilization of different trophic niches and was associated with reproductive isolation within and across lakes. The intralacustrine comparison of whitefish morphs showed that these systems represent two levels of adaptive divergence: (1) a consistent littoral–pelagic resource axis; and (2) a more variable littoral–profundal resource axis. The results also indicate that the profundal whitefish morph has diverged repeatedly from the ancestral littoral whitefish morph in sympatry in two different watercourses. In contrast, all the analyses performed revealed clustering of the pelagic whitefish morphs across lakes suggesting parallel postglacial immigration with the littoral whitefish morph into each lake. Finally, the analyses strongly suggested that the trophic adaptive trait, number of gill rakers, was under diversifying selection in the different whitefish morphs. Together, the results support a complex evolutionary scenario where ecological speciation acts, but where both allopatric (colonization history) and sympatric (within watercourse divergence) processes are involved.

## Introduction

Adaptive radiation, which is the evolutionary divergence of individuals from a single phylogenetic lineage into a variety of adaptive forms (Futuyma 1998), is a widespread phenomenon in several vertebrate taxa including fish, birds, and amphibians (Schluter 2000; Coyne and Orr 2004; Losos 2010). Adaptive radiation may evolve through divergent selection for alternate niches driven by, for example, resource competition, where reproductive isolation may evolve as a by-product of adaptation to divergent niches and may thus lead to new species formation via ecological speciation process (Schluter 2000; Rundle and Nosil 2005; Schluter and Conte 2009). The whole temporal process of population divergence can be viewed as a continuum of increasingly discrete variation from individual variation in panmictic populations without reproductive isolation, to population-wide phenotypic and genotypic polymorphisms with increasing level of reproductive isolation, which ultimately may lead to speciation (Hendry 2009). Such “speciation continua” appear to be especially common in northern polymorphic freshwater fish species (e.g., Skúlason et al. 1999; Taylor 1999; Schluter 2000; Bernatchez 2004; Klemetsen 2010).

Polymorphic populations are common in several freshwater fish species in the northern hemisphere providing an excellent opportunity to study adaptive radiation and ecological speciation. Northern postglacial lakes are young and represent discrete and partly isolated environments. Several fish species inhabiting these lakes have shown rapid and convergent phenotypic divergence and adaptive radiation into multiple ecotypes, morphs, forms, or species (Robinson and Wilson 1994; Taylor 1999; Schluter 2000). The most common ecomorphological divergence among postglacial fishes appears along the littoral–pelagic resource axis (Schluter 2000; Robinson and Parsons 2002; Siwertsson et al. 2010), whereas the third principal lacustrine niche, the profundal zone, has been far less studied. In postglacial lakes, the littoral zone usually provides the most profitable benthic foraging niche for fish with the highest density and biomass of large invertebrate prey (Kahilainen et al. 2003), whereas the pelagic zone is considered to be the second most profitable niche providing small-sized zooplankton prey (e.g., Klemetsen et al. 1989; Kahilainen et al. 2005). These contrasting niches have been shown to promote morphologically bimodal populations that display phenotypic divergence and reproductive isolation (e.g., Lu and Bernatchez 1999; Østbye et al. 2006). Morphological adaptations in the pelagic niche correspond to a pointed head shape, fusiform body shape, and high number of long gill rakers, whereas littoral niche adaptations are related to a subterminal mouth, robust body shape, and low number of short gill rakers (Schluter and McPhail 1992; Bernatchez et al. 1999; Skúlason et al. 1999). The profundal zone is regarded as the least favorable niche due to low temperatures, poor light conditions, and low benthic resource densities mainly consisting of scattered buried small prey (Kahilainen et al. 2003). Morphological adaptations to the profundal zone include large eyes, pronounced subterminal mouth, and very low amount of short and widely spaced gill rakers (Kahilainen and Østbye 2006; Harrod et al. 2010; Kahilainen et al. 2011). Thus, special adaptations in behavioral and morphological traits often accompany the divergent niche utilization in these three principal lake habitats (Kahilainen and Østbye 2006; Knudsen et al. 2006). Indeed, in the polymorphic Arctic charr, *Salvelinus alpinus*, such adaptations have been shown to be heritable (Klemetsen et al. 2002, 2006).

Despite strong support for ecological speciation in recent years (Bernatchez 2004; Rundle and Nosil 2005; Funk et al. 2006; Hendry 2009; Schluter and Conte 2009; Losos 2010), the relative contributions of natural selection driven by ecological divergence or random processes are far from being resolved. An assessment of these contributions can be performed by comparing divergence between variation in phenotypic traits with that of genetic variation at neutral loci (Storz 2002; Raeymaekers et al. 2007; Miner and Kerr 2011). In natural populations, phenotypic divergence between populations can be quantified as *P*_ST_ (e.g., Leinonen et al. 2006; Ramstad et al. 2010), a phenotypic parallel to *Q*_ST_ (Spitze 1993), where the expectation is that phenotypic divergence should be similar to divergence at neutral loci (*F*_ST_) if the traits are evolving neutrally and have an additive genetic basis (Wright 1951). However, *P*_ST_ can only be compared with *F*_ST_ assuming that the total phenotypic variance equals the additive genetic variance and excludes nonadditive genetic and environmental effects (Merilä and Crnokrak 2001; Whitlock 2008). In the face of gene flow, mutation, and genetic drift, the among-population proportion of genetic variance in phenotypic traits is expected to equal that of neutral molecular loci (Lande 1992). Divergent selection may be presumed if divergence in phenotypic traits is stronger than expected from neutral loci (Merilä and Crnokrak 2001; Bernatchez 2004; Whitlock 2008). Accordingly, weaker divergence in phenotypic traits than expected from neutral loci is indicative of stabilizing selection.

**Figure 1:**
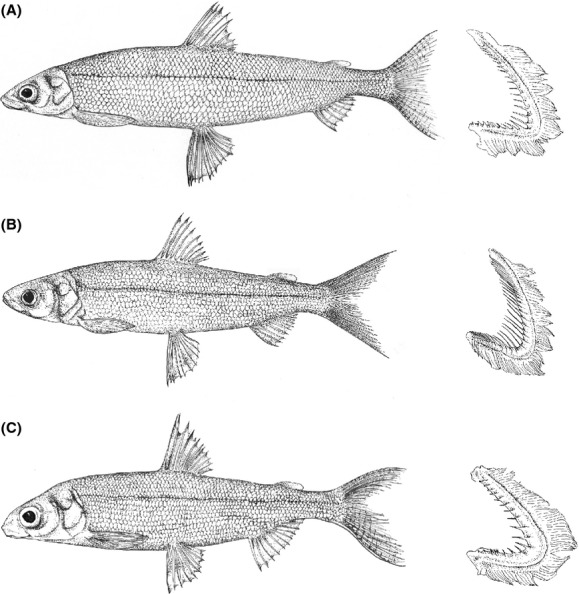
Three morphs of European whitefish (*Coregonus lavaretus* L.) from northern Norway. The gill arches from the ancestral large sparsely rakered (A), and the derived densely rakered (B), and small sparsely rakered (C) whitefish are also shown (line drawings modified from Harrod et al. 2010).

Coregonid fishes have a circumpolar distribution with frequent co-occurrence of multiple ecologically and morphologically distinct morphs (Bernatchez 2004). Genetic and ecological inferences suggest that adaptive radiation is a likely explanation for the polymorphism observed in whitefish (Østbye et al. 2006; Hudson et al. 2007), where also secondary contact of allopatrically evolved lineages contributes to phenotypic diversity in sympatry as seen in the North American coregonids (Bernatchez et al. 2010). According to Schluter (2000), there are four criteria including common ancestry, phenotype-environment correlation, trait utility, and rapid speciation that must be met to support a scenario of adaptive radiation. Indeed, these criteria appear to be met in the European whitefish (*Coregonus lavaretus* [L.]), hereafter referred to as whitefish (e.g., Svärdson 1952, 1979; Amundsen et al. 2004; Bernatchez 2004; Østbye et al. 2005a; Kahilainen et al. 2011; Fig. 1). In northern Fennoscandia, whitefish have diverged into distinct morphs adapted to the three principal habitats, such as the littoral, pelagic, and profundal (Kahilainen and Østbye 2006; Harrod et al. 2010; Siwertsson et al. 2010). The morphs have traditionally been identified based on gill raker number, which is a heritable and ecologically important trait (Bernatchez 2004; Rogers and Bernatchez 2007; Kahilainen et al. 2011), and comprise the large sparsely rakered (LSR), the densely rakered (DR), and the small sparsely rakered (SSR) whitefish morphs (Kahilainen et al. 2004; Siwertsson et al. 2010). Former genetic studies using six microsatellites suggested a scenario of rapid postglacial ecological speciation as the likely explanation for the two most commonly co-occurring whitefish morphs (LSR and DR), originating locally from a process of parallel evolution (Østbye et al. 2006). In other words, it is expected that LSR whitefish in northern Fennoscandia should have a common ancestral origin. Furthermore, it has been suggested that DR and SSR whitefish are postglacially derived morphs following colonization and subsequent sympatric divergence from the ancestral LSR whitefish (Kahilainen and Østbye 2006; Østbye et al. 2006; Harrod et al. 2010). However, the origin of the sympatric whitefish morphs, and the main mechanisms behind genetic diversification and reproductive isolation within and among systems, still remain intriguing evolutionary questions. To elucidate these questions, we sampled three postglacial lake systems inhabited by trimorphic whitefish populations. The main objectives of our study were as follows: firstly, to estimate the level of reproductive isolation among the three whitefish morphs within and across lakes as well as within and between river drainages, and secondly, to contrast the level of reproductive isolation with observed morphological trait divergence (i.e., gill raker number) in the trimorphic lakes. Thus, using the framework of ecological speciation theory, we aimed to examine mechanisms behind morphological divergence and genetic diversification in these trimorphic lakes. We predicted that the following: (1) phenotypic divergence between the three whitefish morphs is positively associated with the utilization of different niches within and across lakes; (2) reproductive isolation is found between all sympatric whitefish morphs within and across lakes; (3) the level of reproductive isolation among whitefish morphs is associated with adaptive trophic traits (i.e., gill raker number) and niche utilization, implying local adaptation with subsequent accumulation of reproductive isolation and finally, (4) similar evolutionary processes have occurred in each of the three lakes, supporting a possible scenario of parallel incipient ecological speciation.

**Figure 2:**
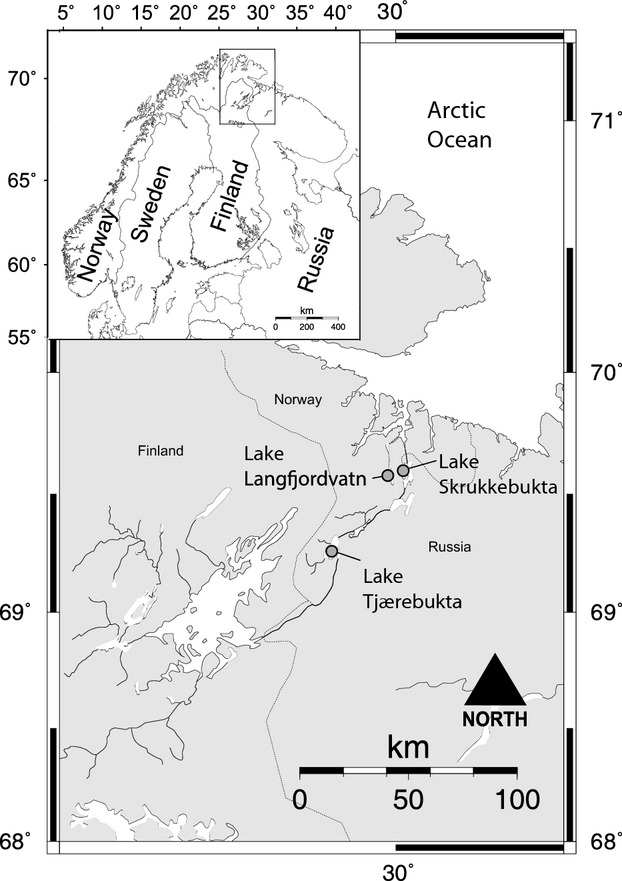
The locations of the study lakes in northern Fennoscandia.

The three study lakes (Lake Langfjordvatn, Lake Skrukkebukta, and Lake Tjærebukta, Fig. 2) were formed during the late Pleistocene in the period of extensive deglaciation of the northern Fennoscandia 10,000–11,000 years before present (ybp; Sollid et al. 1973; Mangerud et al. 2004; Svendsen et al. 2004). The geological history of this area shows that Lake Langfjordvatn appeared at 9700–9200 ybp as a part of the outflow drainage from a major ice-dammed lake (partly the present day L. Inarijärvi, Finland; Kujansuu et al. 1998). The lake discharge to the Arctic Ocean changed at approximately 9000 ybp to follow the present River Pasvik valley isolating L. Langfjordvatn from the present day watercourse (Kujansuu et al. 1998). The geological history of L. Tjærebukta and L. Skrukkebukta is unknown, but as they are located within the R. Pasvik, approx. 100 km apart, it is assumed that they appeared around the time the river was formed. L. Tjærebukta and L. Skrukkebukta are in present day separated by two dam constructions ruling out any major contemporary gene flow between them. Thus, L. Langfjordvatn and the lakes from R. Pasvik represent replicated trimorphic whitefish systems. The lakes are oligotrophic, well oxygenated throughout the year, and have relatively equal proportion of principal niches (littoral, pelagic, and profundal zones). The climate in the region is subarctic and the ice-free season normally ranges from May/June to November. Whitefish is the dominant fish species in the lakes (Table 1), but 6–8 other species have also been recorded, the most common being perch (*Perca fluviatilis* L.), pike (*Esox lucius* L.), burbot (*Lota lota* [L.]), and vendace (*Coregonus albula* [L.]).

**Table 1:** Geographical location and summary of samples with phenotypic and genotypic statistics.

Lake	Location	Morph	Code	Sampling year	*N*	Ls (km^2^)	Ld (m)	Fsp	Mean gill rakers ± SD (minimum-maximum)	H_e_	H_o_	HWE	N_A_	N_AP_
Tjærebukta	69 12′47″N 29 10′20″E	LSR	TbL	2005^a^, 07^b^, 08^a^	39/29	5.1	26	8	24.1 ± 0.3 (19–29)	0.640	0.634	0.622	6.66	0.11
		DR	TbD	2005^a^, 07^b^, 08^a^	45/34				34.5 ± 0.3 (29–40)	0.640	0.620	0.698	7.05	0.13
		SSR	TbS	2005^a^, 07^b^, 08^a^	25/25				20.7 ± 0.4 (18–24)	0.581	0.562	0.358	6.76	0.13
Skrukkebukta	69 33′17″N 30 6′5″E	LSR	SbL	2005^b^, 08^a^, 10^a^	62/35	6.9	38	8	24.0 ± 0.5 (19–29)	0.627	0.601	0.099	7.08	0.17
		DR	SbD	2005^b^, 08^a^^,^^b^, 10^a^	41/57				33.4 ± 0.6 (30–42)	0.664	0.634	0.174	7.03	0.16
		SSR	SbS	2005^b^, 08^a^, 10^a^	61/30				19.9 ± 0.3 (15–25)	0.590	0.565	0.499	6.74	0.20
Langfjordvatn	69 32′6″N 29 58′1″E	LSR	LfL	2007^b^, 08^a^, 09^a^, 10^a^	69/21	2.8	53	6	24.7 ± 0.4 (20–29)	0.649	0.666	0.863	7.05	0.16
		DR	LfD	2007^b^, 08^a^, 09^a^, 10^a^	65/32				35.1 ± 0.9 (29–40)	0.639	0.620	0.785	6.77	0.12
		SSR	LfS	2007^b^, 08^a^^,^^b^, 09^a^, 10^a^	55/38				21.7 ± 0.4 (17–25)	0.636	0.626	0.362	6.56	0.12

Morph, LSR, large sparsely rakered whitefish; DR, densely rakered whitefish; SSR, small sparsely rakered whitefish; Code, sample abbreviations used in text; *N*, sample size for genetic/stable isotope analyses; Ls, lake size; Ld, lake maximum depth; Fsp, number of fish species present; *H*_e_, expected heterozygosity; *H*_o,_ observed heterozygosity; HWE, *P* values of exact tests for deviations from expected Hardy-Weinberg proportions; N_A_, allelic, and N_AP_, private allelic richness.

a Collection of samples for genetic analyses.

b Collection of samples for stable isotope analyses.

## Material and Methods

### Sample collection

In September 2005–2010, we sampled three replicate lakes, Lake Langfjordvatn, Lake Skrukkebukta, and Lake Tjærebukta, located in the northern Fennoscandia (Fig. 2, Table 1). Each lake has relatively equal distribution of deep and shallow areas defined as three principal niches: the littoral zone (<10 m; >1% of light at surface), the profundal zone (>10 m; <1% of light at surface), and the pelagic zone (0–6 m). Fish sampling was performed in all lake habitats many times (i.e., >3 times per habitat) in each year with standardized gillnets consisting of eight 5 m sections with different mesh sizes, 10, 12.5, 15, 18.5, 22, 26, 35, and 45 mm, knot to knot. Benthic gillnets (1.5 m high) were used in the littoral and profundal zones, whereas the pelagic habitat was sampled using 6-m-high floating nets.

### Phenotypic analysis

Morph assignment of the fish was performed in the field, where each individual was classified into LSR, SSR, or DR whitefish according to appearance, head, and body form and a visual evaluation of the gill raker morphology (Amundsen et al. 2004; Kahilainen and Østbye 2006). SSR whitefish is characterized by large eyes, large head, pronounced subterminal mouth, reddish fins, and very short and widely spaced gill rakers (Kahilainen and Østbye 2006; Siwertsson et al. 2010). LSR whitefish are larger in size with silvery sides, dark back, and fins, and robust gill rakers with intermediate length and spacing, whereas the DR whitefish is usually smaller sized, darker colored than LSR whitefish, has pointed head shape and has the largest number of long, thin, and densely packed gill rakers (Harrod et al. 2010; Siwertsson et al. 2010). The number of gill rakers is a good proxy for the phenotypic differences between the whitefish morphs (Amundsen 1988; Kahilainen and Østbye 2006), and in the laboratory, the gill raker number on the first left branchial arch was therefore counted under a dissecting microscope. Number of gill rakers between whitefish morphs in each lake was compared using analyses of variance (ANOVA) and subsequent *post hoc* Tukey′s pairwise tests in the statistical software R (R Core Development Team 2011).

Individual samples of gill filaments were collected from the same individuals as used for phenotypic analysis and preserved in 96% ethanol and stored in −20°C for later molecular analysis.

### Isotope analysis

Stable isotopes of δ^13^C and δ^15^N were used to distinguish trophic resources from the littoral, pelagic, and profundal lake habitats (Vander Zanden and Rasmussen 1999; Syväranta et al. 2006; Harrod et al. 2010). The difference in δ^13^C transfers from pelagic phytoplankton which are δ^13^C depleted compared with benthic algae (Hecky and Hesslein 1995; Vander Zanden and Rasmussen 1999). Profundal organisms are generally enriched in δ^15^N and are together with pelagic organisms also depleted in δ^13^C relative to samples of littoral organisms (Vander Zanden and Rasmussen 1999; Syväranta et al. 2006). Profundal habitats are often dominated by the detritus food chain, which gives more enriched δ^15^N due to the accumulation of the heavier isotope in consumers compared with their prey (e.g., Vander Zanden and Rasmussen 1999; Post 2002). δ^13^C generally increases by 0.4 ± 1.3 and δ^15^N by 3.4 ± 1.0 for each trophic level (Post 2002).

To verify between habitat differences in isotope ratios within each of the three lakes, we sampled and analyzed invertebrate prey from the three principal habitats: a bulk sample of zooplankton (Cladoceran and Copepoda) from the pelagic habitat, semibenthic zooplankton (*Eurycercus lamellatus*) and insect larvae (Trichoptera and Ephemeroptera) from the littoral, and chironomid larvae from the profundal habitat. Invertebrates were analyzed as bulk samples of whole organisms. The differences in prey isotope ratios are expected to be reflected in their fish predators if individuals tend to rely mainly on prey from only one of the habitats. A sample of white muscle tissue (excluding skin, scales, and bones) was taken from below the dorsal fin from each individual fish for stable isotope analysis. Carbon and nitrogen stable isotope ratios from fish muscle tissue reflect the assimilated food sources over several months, typically reflecting the summer growth period in temperate lakes (Perga and Gerdeaux 2005; Buchheister and Latour 2010). Samples for isotope analyses were oven-dried at 60°C for 48 h and then ground to powder using a mortar and pestle. Analysis of carbon (δ^13^C) and nitrogen (δ^15^N) stable isotope ratios was performed at the Institute for Environmental Research, University of Jyväskylä (Finland) using a FlashEA 1112 elemental analyser coupled to a Thermo Finnigan DELTA^plus^ Advantage mass spectrometer (Thermo Fisher Scientific, Waltham, MA). Isotope ratios are expressed as parts per thousands (‰) delta (δ) values relative to the international standards of Vienna Pee Dee Belemnite (for carbon) and atmospheric N_2_ (for nitrogen). Lipids are depleted in δ^13^C (DeNiro and Epstein 1977), and variation in lipid content between whitefish morphs could influence our results. Analyses of C:N contents, which is positively correlated to lipid content, indicated that lipid correction was not necessary (Kiljunen et al. 2006). Stable isotope values of the different whitefish morphs in each lake were compared using ANOVA and *post hoc* Tukey's pairwise tests in the statistical software R (R Core Development Team 2011).

### Microsatellite DNA amplification and analysis

Genomic DNA was extracted from gill filaments by a proteinase K/salt-extraction protocol (Aljanabi and Martinez 1997), modified for extraction using 96-well or by E-Z96 Tissue DNA Kit (OMEGA Bio-tek, Norcross, GA) following the manufacturer instructions. A total of 17 microsatellite loci, BFRO-018 (Susnik et al. 1999), BWF1, BWF2 (Patton et al. 1997), C2–157 (Turgeon et al. 1999), Cla-Tet01, Cla-Tet03, Cla-Tet06, Cla-Tet09, Cla-Tet13, Cla-Tet15, Cla-Tet18 (Winkler and Weiss 2008), Cocl-lav04, Cocl-lav06, Cocl-lav10, Cocl-lav18, Cocl-lav27, Cocl-lav49 (Rogers et al. 2004; Table S1), were amplified in four PCR multiplexes in 2.5 μL reactions following the protocol by Præbel et al. (2013). The fluorescent-labeled PCR products were separated on an ABI 3130 XL Automated Genetic Analyzer (Applied Biosystems, Foster City, CA).

### Genotyping, validation, and quality control of genotypic data

The alleles were scored, and each genotype was automatically binned in predefined allelic bins by the GeneMapper 3.7 software (Applied Biosystems) and verified twice by visual inspection. After the first validation of the genotypes, 9–20% of the individuals within each lake and morph were re-extracted and rerun at all 17 loci to ensure properly amplified alleles, which is reducing the possibility for methodological errors such as large allele drop-out and stutter scoring (but see Pompanon et al. 2005). In order to minimize PCR-introduced errors samples showing low amplification due to low DNA quality or quantity were rerun undiluted or were re-extracted and rerun. In addition, the genotypes resulting from the initial run and the rerun of low amplification samples were manually compared for all individuals to rule out mis-scoring of alleles. If any doubt occurred in this comparison, the samples were re-extracted and rerun at all loci to obtain a consensus genotype. No mismatch was identified in the dataset. The samples were finally screened for abnormalities (null alleles, scoring errors, etc.) in the software MICRO-CHECKER 2.2.3 (Van Oosterhout et al. 2004), using 1000 bootstraps to generate the expected homozygote and heterozygote allele size difference frequencies.

### Genetic diversity and variation

Genetic diversity was quantified by expected (*H*_e_) and observed (*H*_o_) heterozygosity and allelic and private allelic richness. To account for the effect of different sample sizes in the estimate of allelic richness, the rarefaction procedure as implemented in the software HP-RARE 1.0 (Kalinowski 2005) was utilized using the smallest sample (50 genes). *H*_e_ and *H*_o_ as well as deviations from linkage equilibrium (LE) and Hardy–Weinberg equilibrium (HWE) were estimated using GENEPOP 4.0 (Rousset 2007). The tables of pairwise *P* values from the LE and HWE tests were corrected for multiple comparisons by sequential Bonferroni corrections following Rice (1989).

### Genetic divergence among whitefish morphs

Intra- and interlacustrine genetic divergence among the whitefish morphs was tested by principal component analysis (PCA) of allele frequencies. This method is independent of the assumptions of HWE and LE between loci and hence should provide unbiased output of data. The ordination of the morphs was performed in the program PCA-GEN 1.2.1 (Goudet 1999), and the axes tested for significance by 10,000 permutations.

In addition, we tested the genetic divergence as well as evolutionary signatures of parallelism using an unrooted neighbor-joining (NJ) tree of Nei's standard genetic distance (*D*_S_; Nei 1987). The tree was constructed, and the consistency of the tree topology tested by 1000 bootstraps using the program POPULATIONS (Langella 2005) and visualized in TREEVIEW (Page 1996). The tree was combined with a hierarchical approach (Vähä et al. 2007) for inference of population structure using the Bayesian clustering method of STRUCTURE 2.3.2 (Pritchard et al. 2000; Hubisz et al. 2009). We used a model assuming admixture and correlated allele frequencies between *K* population groups (varying burn-ins of 100,000–500,000 replications and 150,000–2,000,000 MCMC replicates). The number of iterations required for the runs to converge tended to increase with decreasing genetic divergence between presumed populations (i.e., decreasing number of *K*). Sampling locations were used as prior information to assist the structuring (the LOCPRIOR model) as recommended for weak signals of structuring (Hubisz et al. 2009). All runs were replicated ten times at each *K* to confirm consistency of log-likelihood probabilities. The most likely (highest ln Pr(*Χ*|*Κ*)/Δ*K* [Evanno et al. 2005]) grouping was found using STRUCTURE HARVESTER (Earl and vonHoldt 2012). For the first run (*K* = 1–12), we used the conservative evaluation of Δ*K*, whereas the evaluation of the most likely structuring in the subsequent rounds was based on the highest ln Pr(*Χ*|*Κ*). In addition, we also examined the proportion of membership of each predefined population in each of the clusters to adjudicate the correct *K*, using *q* value thresholds of 0.25/0.75 (Vähä and Primmer 2006; Vähä et al. 2007; Warnock et al. 2010). The outputs were visualized in DISTRUCT (Rosenberg 2004).

### Differentiation between random genetic drift and selection for the variation in gill raker number

For the present dataset, the number of gill rakers represents the only phenotypic trait that can be directly coupled to individual genotypes. This trait has been reported to be highly heritable (Svärdson 1979; Rogers and Bernatchez 2007), as well as suggested to be influenced by natural selection in both European whitefish (Østbye et al. 2005b) and lake whitefish (*Coregonus clupeaformis*; Bernatchez 2004). We analyzed the data using a joint estimation of neutral genetic differentiation and quantitative genetic differentiation, and their comparison, as described by Ovaskainen et al. (2011). Briefly, this method is based on the observation that if the phenotypic differentiation is a result of random genetic drift, the vector of population means *a*^P^ is statistically distributed as


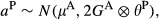


where μ^A^ is the ancestral mean (same for all populations), *G*^A^ is the ancestral matrix of additive genetic variances and covariances (in the present case just variance, as we consider a single trait), θ^P^ is the population-to-population coancestry matrix, and ⊗ denotes the Kronecker product, forming a block matrix based on the elements of *G*^A^ and θ^P^. The observed pattern of population means is compared with the above neutral expectation by using the probability density of the multivariate distribution as an integrative test statistic.

We estimated the pattern of neutral genetic differentiation (θ^P^) from marker data with an admixture F-model (Karhunen and Ovaskainen 2012). We then estimated *a*^P^, μ^A^, and *G*^A^ (and refined the estimate of θ^P^) using the phenotypic data and tested whether the observed divergence in population means was compatible with the expectation from random drift with the method of Ovaskainen et al. (2011). The analyses were performed with the R-package driftsel (Karhunen et al. 2013).

Whether the population genetic structure was more influenced by physical proximity or by habitat types was estimated by comparing the levels of coancestry within lakes (but among all habitats) to those within habitats (but among all lakes). To do so, we investigated how the coancestry coefficients 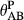 (and thus neutral genetic diversity) between populations A and B were affected by the similarity of habitat and lake. We calculated the posterior distributions of three summary statistics: average coancestry within habitats


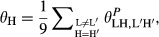


average coancestry within lakes


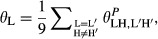


and average coancestry between lakes and habitats


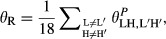


Above, the subscript LH denotes an arbitrary lake-habitat pair, and the denominators 9 and 18 are specific to this study design. Out of the posteriors of θ_H_, θ_L_, and θ_R_, we calculated the following statistics: the posterior probability of a habitat effect *P*(θ_H_ > θ_R_), the posterior probability of a lake effect *P*(θ_L_ > θ_R_), and the posterior probability that the habitat effect is stronger than the lake effect *P*(θ_H_ > θ_L_).

## Results

### Phenotypic analysis

**Figure 3:**
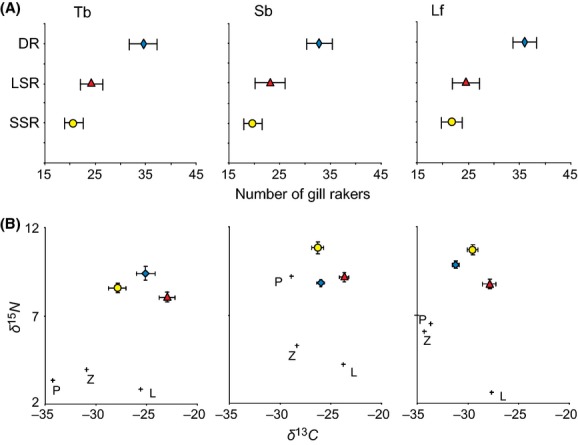
Phenotypic and niche differences among large sparsely rakered whitefish (red triangles), small sparsely rakered whitefish (yellow circles), and densely rakered whitefish (blue diamonds) in Lake Tjærebukta (Tb), Lake Skrukkebukta (Sb), and Lake Langfjordvatn (Lf). Panel (A) shows the mean (±SD) gill raker number panel (B) the mean (±SD) stable isotope ratios of δ^13^C and δ^15^N. The mean isotopic values of putative invertebrate prey for whitefish are marked with L, Z, and P denoting littoral, pelagic, and profundal prey items.

The average number of gill rakers was significantly different among three morphs in all lakes (Table 1, ANOVA: L. Langfjordvatn: *F*_2,101_ = 358.7, *P* < 0.001, L. Skrukkebukta: *F*_2,95_ = 192.1, *P* < 0.001, and L. Tjærebukta: *F*_2,143_ = 415.9, *P* < 0.001; all Tukey′s pairwise tests *P* < 0.001). In all three lakes, the DR whitefish had the highest, LSR whitefish intermediate, and SSR whitefish the lowest number of gill rakers (Fig. 3A).

### Isotope analysis

In all lakes, there were differences in the average stable isotope values (δ^13^C and δ^15^N) among the three whitefish morphs (Fig. 3B). Within each lake, the pattern of differences in stable isotope values among morphs matched the corresponding pattern in prey values from the different habitats (Fig. 3B). The LSR whitefish had higher δ^13^C ratios compared with SSR and DR whitefish in all lakes (ANOVA: L. Langfjordvatn: *F*_2,88_ = 43.5, *P* < 0.001, L. Skrukkebukta: *F*_2,119_ = 32.1, *P* < 0.001, L. Tjærebukta: *F*_2,85_ = 32.1, *P* < 0.001; all Tukey′s pairwise tests *P* < 0.01 except DR-SSR in L. Skrukkebukta: *P* = 0.58). The δ^15^N values were significantly different among the morphs in all three lakes (ANOVA: L. Langfjordvatn: *F*_2,88_ = 61.7, *P* < 0.001, L. Skrukkebukta: *F*_2,119_ = 89.0, *P* < 0.001, and L. Tjærebukta: *F*_2,85_ = 22.9, *P* < 0.001; all Tukey′s pairwise tests *P* < 0.05). The SSR whitefish were more enriched in δ^15^N compared with the LSR morph in all lakes and had the highest δ^15^N values of all morphs in L. Langfjordvatn and L. Skrukkebukta (Fig. 3B).

### Genotyping, validation, and quality control of genotypic data

We did not identify any mismatch between the original individual multilocus genotypes and the re-extracted 9–20% replicates within the present dataset. Heterozygote deficits were indicated by MICRO-CHECKER at three loci in four populations (Cla-Tet13 (SbS), Cocl-lav06 (SbL, SbS, TbS), and Cla-Tet15 [TbD]). In the HWE test, only the loci Cla-Tet13 (SbS) and Cocl-lav06 (SbL) deviated from the expected values and none of them remained significant after sequential Bonferroni corrections. Given the relatively minimal deviations in the MICRO-CHECKER and HWE tests and the independence across populations, we maintained all loci in the subsequent analysis to ensure statistical power.

### Genetic diversity and variation

Summary statistics for the microsatellite loci are listed in Table S1. Number of alleles per locus ranged from 3 in Cocl-lav10 and Cocl-lav18 to 30 in Cla-Tet06. None of the sampled populations showed significant departures from the HWE expectations (Table 1), whereas 10 of 153 individual locus tests displayed significant deviation from HWE. However, all returned nonsignificant after sequential Bonferroni corrections (Table S1). Deviations from linkage equilibrium were only identified within the DR whitefish in L. Langfjordvatn (Cocl-lav06 & BFRO018; Cla-Tet06 & Cla-Tet09) and in the LSR whitefish in L. Tjærebukta morph between the Cla-Tet03 and Cocl-lav06 loci.

All populations of the SSR whitefish showed slightly lower genetic diversity in all diversity estimates compared with the LSR and DR whitefish (Table 1). The LSR and DR whitefish did not differ in genetic diversity in any of the studied lakes.

### Genetic divergence among whitefish morphs

**Figure 4:**
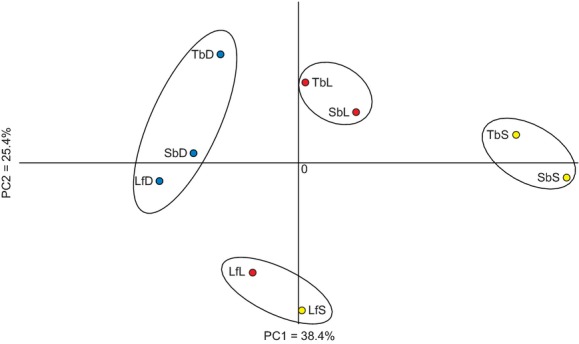
Principal component analysis (PCA) plot of the genetic structuring among whitefish morphs. PC1 and PC2 explain 38.4% and 25.4% of the total variation. The proportion of inertia of both axis returned significant *P* values (*P* < 0.0001). Circles indicate population clusters as identified in the hierarchical STRUCTURE analysis (see Fig. 5). Lake-morph combinations coded as in Table 1 and colored as in Fig. 3.

The allele frequency based principal component analysis (PCA) plot of the genetic structuring among the whitefish morphs revealed groupings of (1) all the DR morphs, (2) the SSR and LSR morphs of L. Langfjordvatn, (3) the SSR morphs across lakes within Pasvik river system, and (4) the LSR morphs across lakes within Pasvik river system (Fig. 4). Each axis, PC1 and PC2, explained 38.4% and 25.4% of the total variation and both axis returned highly significant *P* values (*P* < 0.001).

**Figure 5:**
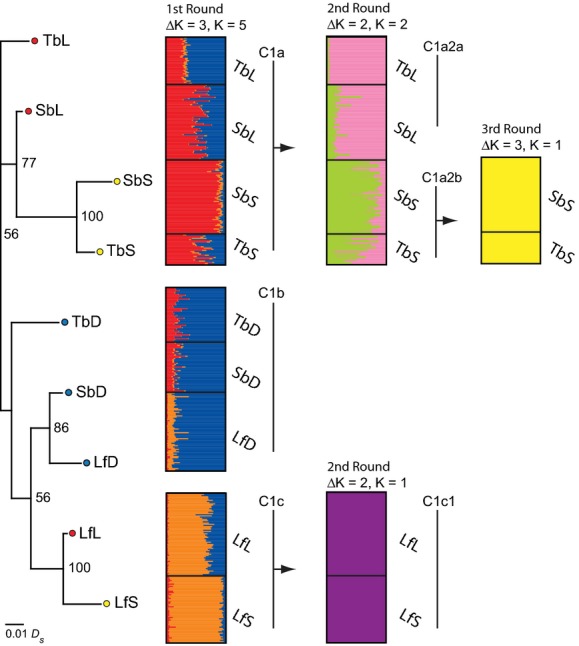
Population structuring among the whitefish morphs in the study lakes. The population structure of densely (D), large sparsely (L), and small sparsely (S) rakered whitefish morphs from L. Langfjordvatn (Lf), L. Skrukkebukta (Sb), and L. Tjærebukta (Tb) was inferred from a combination of a unrooted neighbor-joining (NJ) tree using Nei's standard genetic distance (*D*_S_) and three rounds of hierarchical STRUCTURE analysis. Only bootstrap resampling percentages above 50 are shown for the NJ tree. The colored dots at the branch ends refer to the phenotypic and ecological designation in Fig. 3. In the hierarchical STRUCTURE analysis, black lines separate individuals from different sampling sites (labeled right), and each individual is represented by a thin horizontal line, which is partitioned into *K*-colored segments representing individual's estimated membership fractions in *K* clusters. Vertical black lines delineate the progress of the hierarchical approach, where subsets of the data were subsequently analyzed. For each cluster, absolute values of ln Pr(*X*|*K*) and Δ*K* are plotted for subsequent values of *K* (Fig. S1). The colors used the estimated membership are not related to the colors used for the phenotypic and ecological designation in Fig. 3.

The genetic patterns revealed by the PCA were supported by the hierarchical Bayesian based STRUCTURE analysis in combination with the phylogenetic tree constructed with the drift-based estimator of Nei's standard genetic distance, *D*_S_ (Fig. 5). The first round of STRUCTURE analysis inferred Δ*K* = 3, *K* = 5 for all whitefish morphs (Fig. S1). Using the conservative approach of Evanno et al. (2005), Δ*K* = 3 revealed the clustering C1a: TbL, TbS, SbL, SbS; C1b: TbD, SbD, LfD; and C1c: LfL, LfS (Fig. 5). The subsequent second and third round of analysis of C1a revealed further substructuring into two clusters (Δ*K* = 2, *K* = 2) containing TbL and SbL in one cluster (C1a2a) and TbS and SbS in the other (C1a2b), also supported by the drift-based *D*_S_ tree. Within C1b, all DR populations displayed *q* values above 0.75, providing support for clustering across lakes for this morph, in part supported by the drift-based *D*_S_ tree. C1c consisted of Δ*K* = 2, *K* = 1 with clustering LfL and LfS into one population. A subsequent second round of analysis of this cluster revealed *K* = 1 (C1c1), which is consistent with all other estimates performed herein. To summarize the different approaches, our results indicate that the SSR whitefish may have diverged repeatedly from the LSR whitefish in sympatry in L. Langfjordvatn and in the Pasvik watercourse. In the Pasvik watercourse, there are indications of gene flow between locations for the SSR whitefish and the LSR whitefish. Interestingly, most analyses performed suggested clustering of DR whitefish across watercourses.

### Differentiation between random genetic drift and selection for the variation in gill raker number

**Figure 6:**
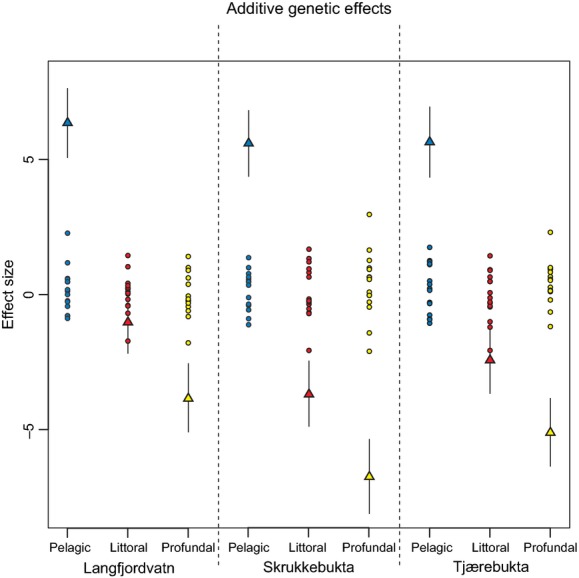
Habitat effects on phenotype. The blue dots represent the pelagic habitat, red the littoral, and yellow the profundal habitats. The columns are Lakes Langfjordvatn (Lf), Skrukkebukta (Sb), and Tjærebukta (Tb). The triangular dots and the error bars represent estimates of population means from the real data (posterior median and 95% credibility interval), whereas the small dots represent the amount of variation expected for a neutral trait (15 replicates over the evolutionary process).

The observed pattern of genetic differentiation measured by the coancestry matrix θ^P^ (Table 2) corresponds to *F*_ST_ = 0.018 (95% credibility interval: 0.016–0.020). Both the division to lakes and the division to habitats had an influence on population structure, as *P*(θ_H_ > θ_R_) > 0.99, and thus average coancestry within lakes was greater than among lakes, and as *P*(θ_L_ > θ_R_) > 0.99, and thus average coancestry within habitats was greater than among habitats. The effect of habitat was stronger than the effect of lake, with *P*(θ_H_ > θ_L_) > 0.99. Phenotypic divergence among the populations and habitats (as measured by *a*^P^) was much greater than what would be expected by the pattern of neutral genetic differentiation (θ^P^) and the estimated amount of additive genetic variation (*G*^A^; Fig. 6). This was also reflected by the formal test of Ovaskainen et al. (2011) which reports a *S*-value of >0.99, thus giving high statistical support for the number of gill rakers being influenced by diversifying selection.

**Table 2:** Posterior median estimate of the coancestry matrix θ^P^.

	LfD	LfL	LfS	SbD	SbL	SbS	TbD	TbL	TbS
LfD	0.024	0.007	0	0.019	0	0	0.010	0	0
LfL	0.007	0.020	0.021	0.005	0.004	0.001	0.002	0.003	0.001
LfS	0	0.021	0.028	0	0.004	0.007	0	0.003	0.004
SbD	0.019	0.005	0	0.018	0.002	0.001	0.012	0.002	0.002
SbL	0	0.004	0.004	0.002	0.017	0.014	0.002	0.015	0.014
SbS	0	0.001	0.007	0.001	0.014	0.036	0.001	0.005	0.026
TbD	0.010	0.002	0	0.012	0.002	0.001	0.023	0.004	0.002
TbL	0	0.003	0.003	0.002	0.015	0.005	0.004	0.020	0.007
TbS	0	0.001	0.004	0.002	0.014	0.026	0.002	0.007	0.023

Lake-morph combinations coded as in Table 1.

## Discussion

This study provides strong evidence supporting resource driven adaptive divergence with subsequent accumulation of reproductive isolation among three whitefish morphs originating from a single ancestral linage. A heritable phenotypic trait associated with trophic performance, the gill raker number, differed significantly between the three whitefish morphs in all lakes. The phenotype-environment association was observed as differences in the resource use, measured using stable isotope values (δ^13^C and δ^15^N) as time-integrated trophic markers. Genetic divergence, despite weak in some comparisons, was found among all whitefish morphs within the three replicated lakes. High statistical support was found for diversifying selection being a major contributor to the variation in the number of gill rakers among the whitefish morphs. Finally, the data suggested repeated sympatric divergence of the SSR whitefish from the LSR whitefish in L. Langfjordvatn and Pasvik watercourse, whereas all analyses performed suggested clustering of DR whitefish across lakes. We discuss these findings below together with their implications for the role of divergent natural selection in adaptive radiation of European whitefish.

In postglacial lakes, the contrasting littoral and pelagic niches have been shown to promote bimodal populations that display large phenotypic divergence and accumulation of reproductive isolation (e.g., Lu and Bernatchez 1999; Østbye et al. 2006). As predicted in the present study, we consistently found intralacustrine reproductive isolation between LSR and DR whitefish. The divergence was also reflected in significant differences in average gill raker numbers between these two morphs and in resource use that is related to the corresponding pattern in prey utilization from the different lake habitats. A similar pattern has also been identified between the LSR and DR whitefish in another study from the region (Østbye et al. 2006). However, this previous study did not investigate lakes harboring SSR whitefish as a sympatric occurring morph specially adapted to live in the profundal habitat. The SSR whitefish significantly diverged in both resource use and in having a significantly lower mean number of gill rakers than the LSR and DR whitefish, in concordance with earlier ecological and morphological studies (Kahilainen and Østbye 2006; Siwertsson et al. 2010; Kahilainen et al. 2011). We also provide the first substantial evidence that divergence in trophic ecology is linked to reproductive isolation between the profundal SSR whitefish morph and its sympatric counterparts. The most pronounced difference in terms of reproductive isolation was found between the SSR and DR whitefish. This is not surprising, given that the SSR whitefish is a profundal soft-bottom foraging specialist suggested to have diverged from the LSR whitefish, while the DR whitefish is a pelagic zooplanktivore specialist (Kahilainen and Østbye 2006; Harrod et al. 2010). The profound genetic difference between the SSR and DR whitefish is also consistent with the ecomorphological divergence observed between these morphs (Harrod et al. 2010; Kahilainen et al. 2011; present results). A recent study investigated the early stage of divergence in resource use, gill raker number, and genetic differentiation in LSR whitefish from the littoral and profundal niches in three lakes in the western part of northern Fennoscandia (Siwertsson et al. 2013). These lakes were colonized approximately 5000 years later than the lakes included in the present study (Sollid et al. 1973), thus representing sympatric systems at a younger stage of divergence (i.e., ecomorphological and genetic divergence less progressed). The study demonstrated that the littoral–profundal axis divergence of the LSR whitefish represented a more recent stage in the process of ecological speciation compared with the benthic–pelagic (i.e., LSR and DR whitefish) resource axis (Siwertsson et al. 2013). The present study demonstrates that a distinct ecomorphological divergence has already been established between the LSR and SSR whitefish, and it was therefore expected that the whitefish morphs have accumulated a higher degree of reproductive isolation. However, the microsatellite analyses revealed varying degrees of reproductive isolation between LSR versus SSR whitefish among the replicate lakes. Moreover, the genetic results also supported origin of the SSR whitefish from the LSR whitefish, thus suggesting that these systems are in a continuous process of divergence along the littoral–profundal resource axis. Hence, our data comply with the findings by Siwertsson et al. (2013) that these northern Fennoscandian whitefish systems represent (at least) two levels of adaptive divergence along a consistent littoral–pelagic resource axis and a more variable littoral–profundal resource axis. Thus as predicted, the repeated intralacustine consistency between resource use and mean number of gill rakers (phenotype–environment correlation) together with our genetic results of reproductive isolation between all three whitefish morphs support previous suggestions that these systems are in a process of incipient ecological speciation.

Phenotype–environment correlations, which is correlation of certain phenotypic traits to specific environment characteristics, are one criteria for adaptive radiation (Schluter 2000). Such a correlation was, as predicted, strongly indicated in the present study as clear associations between gill raker numbers and the utilization of habitat, diet, and isotopic niche of these three sympatric whitefish morphs. Similar relationships have also been observed in earlier whitefish studies (Kahilainen et al. 2004, 2011; Harrod et al. 2010). Svärdson (1979) showed that the gill raker number is a highly heritable trait. The gill raker number also appears to be a key trait in the ecology of coregonids and seems to play a central role in the adaptive diversification process across the distributional range (Bernatchez 2004; Østbye et al. 2005a,b, 2006; Rogers and Bernatchez 2007; Vonlanthen et al. 2009; Kahilainen et al. 2011; Siwertsson et al. 2012). The importance of gill rakers in *Coregonus* is related trait utility via feeding efficiency, where higher numbers of gill rakers facilitate feeding of smaller prey items such as pelagic zooplankton (Kahilainen et al. 2011). Here, divergent selection on the gill raker number should operate in opposite directions in DR and SSR whitefish, where the former benefit from a higher number of gill rakers to feed on small zooplankton prey, while the latter use, their lower number of gill rakers to forage on larger benthic prey from profundal sediments. However, whether the gill raker trait in natural populations of European whitefish is subjected to divergent natural selection, as hypothesized in several recent ecological studies (e.g., Østbye et al. 2005b; Harrod et al. 2010; Siwertsson et al. 2010) has yet to be illuminated in more detail. We found much greater phenotypic divergence among the populations and habitats than expected if the trait variation was driven by neutral genetic differentiation alone. The divergence was also confirmed by the formal test by Ovaskainen et al. (2011), thus giving high statistical support for the gill raker trait being influenced by diversifying selection. The discrepancy between the phenotypic and neutral genetic differentiation was repeatedly found to be the greatest between the most specialized morphs that is the pelagic zooplanktivore DR whitefish and the profundal soft-bottom benthivore SSR whitefish. In Lake Femund, in eastern Norway, Østbye et al. (2005b) found similarly strong support for divergent selection on gill raker number and gill raker length between diverged whitefish morphs based on a *Q*_ST_ − *F*_ST_ comparison. In addition, only minor differences were observed in these two traits (i.e., *F*_ST_ = *Q*_ST_ or *F*_ST_ > *Q*_ST_) when comparing geographical replicates within the four studied whitefish morphs. In the north American lake whitefish, the gill raker numbers of the sympatric normal and dwarf morphs have been shown to be the only trait out of eight meristic and 18 morphological characters that were consistently under the influence of divergent selection (Bernatchez 2004). When addressing the differential influence of divergent selection on the gill raker number, the study only found a significant contribution within the dwarf morph. Our results suggest that isolation by adaptation (Nosil et al. 2009), which is the fact that similar genetic material is likely to be conserved in populations living in similar habitats, may be an important mechanism in these northern postglacial systems. Future studies may therefore beneficially utilize this watercourse in order to disentangle the role of the gill raker trait in the speciation process of whitefish morphs.

The differences observed in *F*_ST_ − *P*_ST_ comparison should be interpreted with caution as they are obtained from natural populations and not from a controlled experimental facility. Like most *F*_ST_ − *Q*_ST_ comparisons (see e.g., Merilä and Crnokrak 2001), the method by Ovaskainen et al. (2011) assumes that environmental effects would be independent of the rearing environment and thus the phenotypes would directly reflect the additive genetic effects. Typically, these conditions are only met in a common-garden design. In this study, the phenotypes were measured in the wild, so that it is possible that the pattern of phenotypic differentiation, to some degree, results from phenotypic plasticity. A formal cross-transplant experiment would therefore reveal the robustness of the test within these whitefish systems. In addition, valuable insights may also be gained by comparing systems of mono-, di-, and trimorphic whitefish populations, as varying degrees of phenotypic and genotypic variation is likely as observed also in this study.

Our results support an evolutionary scenario of sympatric divergence for the three whitefish morphs as previously suggested by Østbye et al.'s (2005a, 2006) studies of the LSR and DR whitefish pair. This scenario is especially convincing for the origin of the SSR whitefish. This whitefish morph has not been identified in other large watercourses within the region *per se* (but see: Siwertsson et al. 2010). Our results show that the SSR whitefish probably has diverged repeatedly from the LSR whitefish in sympatry both in L. Langfjordvatn and in Pasvik watercourse. Additionally for the Pasvik watercourse, our results reveal that the SSR whitefish (SbS and TbS) likely have a joint ancestry as they are clustered together in all analyses. This inference is in agreement with our intralacustrine comparison, where divergence in ecomorphology was followed by a large degree of reproductive isolation between the LSR and SSR whitefish in the Pasvik watercourse. In L. Langfjordvatn, in contrast, only weak reproductive isolation was identified although a similar resource use and ecomorphological divergence were found to be established. The separate clustering of the SSR whitefish in Pasvik and L. Langfjordvatn from a monophyletic lineage, taken together with the varying degrees of reproductive isolation despite ecomorphological divergence, shows that these systems might represent different stages in the process of ongoing sympatric divergence (see e.g., Hendry 2009). Interestingly, all analyses performed herein suggest a clustering of the DR whitefish across lakes rather than within lakes, which suggest that this morph may have an allopatric origin colonizing different lakes immediately after glacier retreat. The use of such proglacial lakes as stepping stones for postglacial colonization has also been observed in north American ciscoes (Turgeon and Bernatchez 2003). Hence, alternative explanations to the repeated sympatric origin of the DR whitefish from the LSR whitefish as suggested by Østbye et al. (2006) could, for example, be a parallel postglacial colonization of these whitefish morphs or a sympatric divergence upon secondary contact of the same morph from repeated colonizations as observed in lake whitefish (Pigeon et al. 1997; Lu et al. 2001). However, further studies, covering a larger geographical area, are needed to shed light over these alternative scenarios of the possible origin of these sympatric whitefish morphs.

In conclusion, we found varying levels of reproductive isolation among the three whitefish morphs within and across lakes as well as within and between two watercourses. The reproductive isolation was related to a consistent ecomorphological divergence as inferred by gill raker numbers and niche utilization in the present trimorphic parallel lake systems. Finally, the adaptive trophic trait, gill raker number, was shown to be influenced by diversifying selection in all the whitefish morphs probably promoting and maintaining the early divergence and subsequent accumulation of reproductive isolation among the whitefish morphs. Thus, among the many potential theoretical scenarios of divergence, our data currently support replicated events of ecological speciation, but where both allopatric and sympatric forces may interact. Here, allopatric processes can be defined as immediate colonization of proglacial lakes by DR and LSR whitefish. Later in more isolated postglacial lakes, sympatric divergence of SSR whitefish to underutilized profundal niche from littoral dwelling LSR whitefish would occur via ecological opportunity. The investigated lakes provide an excellent platform for further investigations of mechanisms involved in on-going sympatric divergence such as the influence of contemporary gene flow on maintenance of reproductive barriers within and among whitefish morphs, association of functional adaptive morphology to the underlying genes and the interplay of phenotype and genotype correlations with environment at the individual level – all leading to a more comprehensive understanding of the early processes of ecological speciation. Evidently, illuminating responsible mechanisms behind the early steps of species formation, being allopatric or sympatric, is a daunting task for evolutionary biologists. Given that neutral loci may float freely among divergent populations while loci influenced by natural selection may not (e.g., Renaut et al. 2012), a follow-up study using next generation-sequence technology to obtain genome wide coverage of single nucleotide polymorphisms (SNP's) should be performed to contrast divergence at neutral and selected loci, to pinpoint candidate loci influence by selection and to address the evolutionary origin of the sympatric morphs in more lakes than included in the present study.

## Supporting Information

**Table S1.** Summary statistics for each locus and population across the three whitefish (*Coregonus lavaretus* L.) morphs from three localities in northern Norway.

**Figure S1.** Summary of results from STRUCTURE for inferring K populations among the tested whitefish (*Coregonus lavaretus* L.) populations.

## References

[b1] Aljanabi SM,, Martinez I (1997). Universal and rapid salt-extraction of high quality genomic DNA for PCR-based techniques. Nucleic Acids Res.

[b2] Amundsen P-A (1988). Habitat and food segregation of two sympatric populations of whitefish (*Coregonus lavaretus* L. s.l) in Stuorajavri, northern Norway. Nord. J. Freshw. Res.

[b3] Amundsen P-A, Bøhn T, Våga GH (2004). Gill raker morphology and feeding ecology of two sympatric morphs of European whitefish (*Coregonus lavaretus*. Ann. Zool. Fenn.

[b4] Bernatchez L, Hendry AP, Stearns SC (2004). Ecological theory of adaptive radiation. An empirical assessment from coregonine fishes (Salmoniformes). Evolution illuminated, salmon and their relatives.

[b5] Bernatchez L, Chouinard A, Lu GQ (1999). Integrating molecular genetics and ecology in studies of adaptive radiation: whitefish, *Coregonus sp*., as a case study. Biol. J. Linn. Soc.

[b6] Bernatchez L, Renaut S, Whiteley AR, Derome N, Jeukens J, Landry L (2010). On the origin of species: insights from the ecological genomics of lake whitefish. Philos. Trans. R. Soc. Lond. B Biol. Sci.

[b7] Buchheister A,, Latour RJ (2010). Turnover and fractionation of carbon and nitrogen stable isotopes in tissues of a migratory coastal predator, summer flounder (*Paralichthys dentatus*. Can. J. Fish. Aquat. Sci.

[b8] Coyne JA,, Orr HA (2004). Speciation.

[b9] DeNiro MJ,, Epstein S (1977). Mechanism of carbon isotope fractionation associated with lipid-synthesis. Science.

[b10] Earl DA,, vonHoldt BM (2012). STRUCTURE HARVESTER: a website and program for visualizing STRUCTURE output and implementing the Evanno method. Conserv. Genet. Resour.

[b11] Evanno G, Regnaut S, Goudet J (2005). Detecting the number of clusters of individuals using the software STRUCTURE: a simulation study. Mol. Ecol.

[b12] Funk DJ, Nosil P, Etges WJ (2006). Ecological divergence exhibits consistently positive associations with reproductive isolation across disparate taxa. Proc. Natl Acad. Sci. USA.

[b13] Futuyma DJ (1998). Evolutionary biology.

[b14] Goudet J (1999). http://www2.unil.ch/popgen/softwares/pcagen.htm.

[b15] Harrod C, Mallela J, Kahilainen KK (2010). Phenotype-environment correlations in a putative whitefish adaptive radiation. J. Anim. Ecol.

[b16] Hecky RE,, Hesslein RH (1995). Contributions of benthic algae to lake food webs as revealed by stable isotope analysis. J. North Am. Benthol. Soc.

[b17] Hendry AP (2009). Ecological speciation! Or the lack thereof?. Can. J. Fish. Aquat. Sci.

[b18] Hubisz MJ, Falush D, Stephens M, Pritchard JK (2009). Inferring weak population structure with the assistance of sample group information. Mol. Ecol. Resour.

[b19] Hudson AG, Vonlanthen P, Müller R, Seehausen O (2007). Review: the geography of speciation and adaptive radiation in coregonines. Adv. Limnol.

[b20] Kahilainen K,, Østbye K (2006). Morphological differentiation and resource polymorphism in three sympatric whitefish *Coregonus lavaretus* (L.) forms in a subarctic lake. J. Fish Biol.

[b21] Kahilainen K, Lehtonen H, Könönen K (2003). Consequence of habitat segregation to growth rate of two sparsely rakered whitefish (*Coregonus lavaretus* (L.)) forms in a subarctic lake. Ecol. Freshw. Fish.

[b22] Kahilainen K, Malinen T, Tuomaala A, Lehtonen H (2004). Diel and seasonal habitat and food segregation of three sympatric *Coregonus lavaretus* forms in a subarctic lake. J. Fish Biol.

[b23] Kahilainen K, Alajärvi E, Lehtonen H (2005). Planktivory and diet-overlap of densely rakered whitefish (*Coregonus lavaretus* (L.)) in a subarctic lake. Ecol. Freshw. Fish.

[b24] Kahilainen KK, Siwertsson A, Gjelland KØ, Knudsen R, Bøhn T, Amundsen P-A (2011). The role of gill raker number variability in adaptive radiation of coregonid fish. Evol. Ecol.

[b25] Kalinowski ST (2005). HP-RARE 1.0: a computer program for performing rarefaction on measures of allelic richness. Mol. Ecol. Notes.

[b26] Karhunen M,, Ovaskainen O (2012). Estimating population-level coancestry coefficients by an admixture F model. Genetics.

[b27] Karhunen M, Merilä J, Leinonen T, Cano JM, Ovaskainen O (2013). driftsel: an R package for detecting signals of natural selection in quantitative traits. Mol. Ecol. Resour.

[b28] Kiljunen M, Grey J, Sinisalo T, Harrod C, Immonen H, Jones RI (2006). A revised model for lipid-normalizing delta C-13 values from aquatic organisms, with implications for isotope mixing models. J. Appl. Ecol.

[b29] Klemetsen A (2010). The charr problem revisited: exceptional phenotypic plasticity promotes ecological speciation in postglacial lakes. Freshw. Rev.

[b30] Klemetsen A, Amundsen P-A, Muladal H, Rubach S, Solbakken JI (1989). Habitat shifts in a dense, resident Arctic charr *Salvelinus alpinus* population. Physiol. Ecol. Japan.

[b31] Klemetsen A, Elliott JM, Knudsen R, Sørensen P (2002). Evidence for genetic differences in the offspring of two sympatric morphs of Arctic charr. J. Fish Biol.

[b32] Klemetsen A, Knudsen R, Primicerio R, Amundsen P-A (2006). Divergent, genetically based feeding behaviour of two sympatric Arctic charr, *Salvelinus alpinus* (L.), morphs. Ecol. Freshw. Fish.

[b33] Knudsen R, Klemetsen A, Amundsen P-A, Hermansen B (2006). Incipient speciation through niche expansion: an example from the Arctic charr in a subarctic lake. Proc. Biol. Sci.

[b34] Kujansuu R, Eriksson B, Grönlund T (1998). Lake Inarijärvi, northern Finland: sedimentation and late quaternary evolution. Geological Survey of Finland.

[b35] Lande R (1992). Neutral theory of quantitative genetic variance in an island model with local extinction and colonization. Evolution.

[b36] Langella O (2005). http://bioinformatics.org/project/?group_id=84.

[b37] Leinonen T, Cano JM, Mäkinen H, Merilä J (2006). Contrasting patterns of body shape and neutral genetic divergence in marine and lake populations of threespine sticklebacks. J. Evol. Biol.

[b38] Losos JB (2010). Adaptive radiation, ecological opportunity, and evolutionary determinism. Am. Nat.

[b39] Lu G,, Bernatchez L (1999). Correlated trophic specialization and genetic divergence in sympatric lake whitefish ecotypes (*Coregonus clupeaformis*): support for the ecological speciation hypothesis. Evolution.

[b40] Lu G, Basley DJ, Bernatchez L (2001). Contrasting patterns of mitochondrial DNA and microsatellite introgressive hybridization between lineages of lake whitefish (*Coregonus clupeaformis*); relevance for speciation. Mol. Ecol.

[b41] Mangerud J, Jakobsson M, Alexanderson H, Astakhov V, Clarke GKC, Henriksen M (2004). Ice-dammed lakes and rerouting of the drainage of northern Eurasia during the last glaciation. Quatern. Sci. Rev.

[b42] Merilä J,, Crnokrak P (2001). Comparison of genetic differentiation at marker loci and quantitative traits. J. Evol. Biol.

[b43] Miner BE,, Kerr B (2011). Adaptation to local ultraviolet radiation conditions among neighbouring *Daphnia* populations. Proc. Biol. Sci.

[b44] Nei M (1987). Molecular evolutionary genetics.

[b45] Nosil P, Funk DJ, Ortiz-Barrientos D (2009). Divergent selection and heterogeneous genomic divergence. Mol. Ecol.

[b46] Østbye K, Bernatchez L, Næsje TF, Himberg KJM, Hindar K (2005a). Evolutionary history of the European whitefish *Coregonus lavaretus* (L.) species complex as inferred from mtDNA phylogeography and gill-raker numbers. Mol. Ecol.

[b47] Østbye K, Næsje TF, Bernatchez L, Sandlund OT, Hindar K (2005b). Morphological divergence and origin of sympatric populations of European whitefish (*Coregonus lavaretus* L.) in Lake Femund, Norway. J. Evol. Biol.

[b48] Østbye K, Amundsen PA, Bernatchez L, Klemetsen A, Knudsen R, Kristoffersen R (2006). Parallel evolution of ecomorphological traits in the European whitefish *Coregonus lavaretus* (L.) species complex during postglacial times. Mol. Ecol.

[b49] Ovaskainen O, Karhunen M, Zheng C, Arias JMC, Merilä J (2011). A new method to uncover signatures of divergent and stabilizing selection in quantitative traits. Genetics.

[b50] Page RDM (1996). TreeView: an application to display phylogenetic trees on personal computers. Comput. Appl. Biosci.

[b51] Patton JC, Gallaway BJ, Fechhelm RG, Cronin MA (1997). Genetic variation of microsatellite and mitochondrial DNA markers in broad whitefish (*Coregonus nasus*) in the Colville and Sagavanirktok rivers in northern Alaska. Can. J. Fish. Aquat. Sci.

[b52] Perga ME,, Gerdeaux D (2005). ‘Are fish what they eat’ all year round?. Oecologia.

[b53] Pigeon D, Chouinard A, Bernatchez L (1997). Multiple modes of speciation involved in the parallel evolution of sympatric morphotypes of lake whitefish (*Coregonus clupeaformis*, Salmonidae). Evolution.

[b54] Pompanon F, Bonin A, Bellemain E, Taberlet P (2005). Genotyping errors: causes, consequences and solutions. Nat. Rev. Genet.

[b55] Post DM (2002). Using stable isotopes to estimate trophic position: models, methods, and assumptions. Ecology.

[b56] Præbel K, Westgaard J-I, Amundsen P-A, Siwertsson A, Knudsen R, Kahilainen KK (2013). A diagnostic tool for efficient analysis of population structure, hybridization and conservation status of European whitefish (*Coregonus lavaretus* (L.)) and vendace (*C. albula* (L.)). Adv. Limnol.

[b57] Pritchard JK, Stephens M, Donnelly P (2000). Inference of population structure using multilocus genotype data. Genetics.

[b58] R Core Development Team (2011). R: a language and environment for statistical computing.

[b59] Raeymaekers JAM, Larmuseau JKJ, Van Houdt MHD, Geldof S, Volckaert FAM (2007). Divergent selection as revealed by P_ST_ and QTL-based F_ST_ in three-spined stickleback (*Gasterosteus aculeatus*) populations along a coastal-inland gradient. Mol. Ecol.

[b60] Ramstad KM, Woody CA, Allendorf FW (2010). Recent local adaptation of sockeye salmon to glacial spawning habitats. Evol. Ecol.

[b61] Renaut S, Maillet N, Normandeau E, Sauvage C, Derome N, Rogers SM (2012). Genome-wide patterns of divergence during speciation: the lake whitefish case study. Philos. Trans. R. Soc. Lond. B Biol. Sci.

[b62] Rice WR (1989). Analyzing tables of statistical tests. Evolution.

[b63] Robinson BW,, Parsons KJ (2002). Changing times, spaces, and faces: tests and implications of adaptive morphological plasticity in the fishes of northern postglacial lakes. Can. J. Fish. Aquat. Sci.

[b64] Robinson BW,, Wilson DS (1994). Character release and displacement in fishes – a neglected literature. Am. Nat.

[b65] Rogers SM,, Bernatchez L (2007). The genetic architecture of ecological speciation and the association with signatures of selection in natural lake whitefish (*Coregonus* sp Salmonidae) species pairs. Mol. Biol. Evol.

[b66] Rogers SM, Marchand MH, Bernatchez L (2004). Isolation, characterization and cross-salmonid amplification of 31 microsatellite loci in the lake whitefish (*Coregonus clupeaformis*, Mitchill). Mol. Ecol. Notes.

[b67] Rosenberg NA (2004). DISTRUCT: a program for the graphical display of population structure. Mol. Ecol. Notes.

[b68] Rousset F (2007). Genepop'007: a complete reimplementation of the Genepop software for Windows and Linux. Molecular Ecology Resourses.

[b69] Rundle HD,, Nosil P (2005). Ecological speciation. Ecol. Lett.

[b70] Schluter D (2000). The ecology of adaptive radiation.

[b71] Schluter D,, Conte GL (2009). Genetics and ecological speciation. Proc. Natl Acad. Sci. USA.

[b72] Schluter D,, McPhail JD (1992). Ecological character displacement and speciation in sticklebacks. Am. Nat.

[b73] Siwertsson A, Knudsen R, Kahilainen KK, Præbel K, Primicerio R, Amundsen P-A (2010). Sympatric diversification as influenced by ecological opportunity and historical contingency in a young species lineage of whitefish. Evol. Ecol. Res.

[b74] Siwertsson A, Knudsen R, Amundsen P-A (2012). Temporal stability in gill raker numbers of subarctic European whitefish populations. Adv. Limnol.

[b75] Siwertsson A, Knudsen R, Præbel K, Adams CE, Newton J, Amundsen P-A (2013). Discrete foraging niches promote ecological, phenotypic and genetic divergence in sympatric whitefish (*Coregonus lavaretus*. Evol. Ecol.

[b76] Skúlason S, Snorrason SS, Jónsson B, Magurran AE, May RM (1999). Sympatric morphs, populations and speciation in freshwater fish with emphasis on Arctic charr. Evolution of biological diversity.

[b77] Sollid JL, Andersen S, Hamre N, Kjeldsen O, Salvigsen O, Sturød S (1973). Deglaciation of Finnmark, North Norway. Nor. Geogr. Tidsskr.

[b78] Spitze K (1993). Population-structure in *Daphnia obtusa* – quantitative genetic and allozymic variation. Genetics.

[b79] Storz JF (2002). Contrasting patterns of divergence in quantitative traits and neutral DNA markers: analysis of clinal variation. Mol. Ecol.

[b80] Susnik S, Snoj A, Dovc P (1999). Microsatellites in grayling (*Thymallus thymallus*): comparison of two geographically remote populations from the Danubian and Adriatic river basin in Slovenia. Mol. Ecol.

[b81] Svärdson G (1952). The coregonid problem. IV. The significance of scales and gillrakers. Rep. Inst. Freshw. Res. Drottningholm.

[b82] Svärdson G (1979). Speciation of Scandinavian *Coregonus*. Rep. Inst. Freshw. Res. Drottningholm.

[b83] Svendsen JI, Alexanderson H, Astakhov VI, Demidov I, Dowdeswell JA, Funder S (2004). Late quaternary ice sheet history of northern Eurasia. Quatern. Sci. Rev.

[b84] Syväranta J, Hämäläinen H, Jones RI (2006). Within-lake variability in carbon and nitrogen stable isotope signatures. Freshw. Biol.

[b85] Taylor EB (1999). Species pairs of north temperate freshwater fishes: evolution, taxonomy, and conservation. Rev. Fish Biol. Fisheries.

[b86] Turgeon J,, Bernatchez L (2003). Reticulate evolution and phenotypic diversity in North American ciscoes, *Coregonus* ssp. (Teleostei: Salmonidae): implications for the conservation of an evolutionary legacy. Conserv. Genet.

[b87] Turgeon J, Estoup A, Bernatchez L (1999). Species flock in the North American Great Lakes: molecular ecology of Lake Nipigon Ciscoes (Teleostei: Coregonidae: *Coregonus*. Evolution.

[b88] Vähä JP,, Primmer CR (2006). Efficiency of model-based Bayesian methods for detecting hybrid individuals under different hybridization scenarios and with different numbers of loci. Mol. Ecol.

[b89] Vähä JP, Erkinaro J, Niemelä E, Primmer CR (2007). Life-history and habitat features influence the within-river genetic structure of Atlantic salmon. Mol. Ecol.

[b90] Van Oosterhout C, Hutchinson WF, Wills DPM, Shipley P (2004). MICRO-CHECKER: software for identifying and correcting genotyping errors in microsatellite data. Mol. Ecol. Notes.

[b91] Vander Zanden MJ, Rasmussen JB (1999). Primary consumer delta C-13 and delta N-15 and the trophic position of aquatic consumers. Ecology.

[b92] Vonlanthen P, Roy D, Hudson AG, Largiader CR, Bittner D, Seehausen O (2009). Divergence along a steep ecological gradient in lake whitefish (*Coregonus* sp.). J. Evol. Biol.

[b93] Warnock WG, Rasmussen JB, Taylor EB (2010). Genetic clustering methods reveal bull trout (*Salvelinus confluentus*) fine-scale population structure as a spatially nested hierarchy. Conserv. Genet.

[b94] Whitlock MC (2008). Evolutionary inference from Q_ST_. Mol. Ecol.

[b95] Winkler KA,, Weiss S (2008). Eighteen new tetranucleotide microsatellite DNA markers for *Coregonus lavaretus* cloned from an alpine lake population. Mol. Ecol. Resour.

[b96] Wright S (1951). The genetical structure of populations. Ann. Eugen.

