# Supplementary material for: Ecological speciation in postglacial European whitefish: rapid adaptive radiations into the littoral, pelagic, and profundal lake habitats
Source: Ecol Evol. 2013 Nov 11;3(15):4970–86. doi: 10.1002/ece3.867 (PMC3892361; doi:10.1002/ece3.867)
Supplement: Supplementary file 1 [file ece30003-4970-SD1.docx]

**Supporting information**

Table S1. Summary of sample sizes (*N*), allelic richness (NA), private allelic richness (NAP), observed (*H_o_*) and expected (*H_e_*) heterozygosity, *p*-values of tests for deviations from expected Hardy-Weinberg proportions (H.-W. test) of the studied populations. Table-wide significance levels for H.-W. tests were applied for each locus separately (bold values), using the sequential Bonferroni technique (Rice 1989). None of the p-values were significant after sequential Bonferroni correction (at the 5% level or less). For details about loci see Præbel *et al.* (2013).

| **Pop** |  | **BWF1** | **BWF2** | **Cla-Tet03** | **Cla-Tet13** | **Cla-Tet18** | **Cocl-lav04** | **Cocl-Lav06** | **Cocl-Lav10** | **Cocl-lav27** | **BFRO-018** | **Cocl-lav18** | **Cocl-lav49** | **Cla-Tet06** | **Cla-Tet09** | **Cla-Tet15** | **C2-157** | **Cla-Tet01** |
| --- | --- | --- | --- | --- | --- | --- | --- | --- | --- | --- | --- | --- | --- | --- | --- | --- | --- | --- |
| **TbL** | **N** | 39 | 39 | 39 | 39 | 39 | 39 | 39 | 39 | 39 | 39 | 39 | 39 | 39 | 39 | 39 | 39 | 39 |
|  | **NA** | 6.0 | 6.5 | 8.1 | 6.5 | 5.5 | 3.9 | 7.6 | 3.0 | 4.3 | 4.9 | 2.0 | 6.6 | 14.0 | 10.0 | 4.0 | 7.9 | 12.4 |
|  | **NAP** | 0.0 | 0.0 | 0.0 | 0.0 | 0.0 | 0.1 | 0.0 | 0.0 | 0.6 | 0.0 | 0.0 | 0.2 | 0.3 | 0.0 | 0.0 | 0.2 | 0.4 |
|  | **Ho** | 0.897 | 0.769 | 0.692 | 0.744 | 0.359 | 0.333 | 0.821 | 0.590 | 0.179 | 0.462 | 0.385 | 0.769 | 0.897 | 0.795 | 0.667 | 0.821 | 0.923 |
|  | **He** | 0.811 | 0.729 | 0.767 | 0.778 | 0.376 | 0.291 | 0.792 | 0.554 | 0.168 | 0.510 | 0.311 | 0.800 | 0.926 | 0.801 | 0.641 | 0.821 | 0.884 |
|  | **H-W-test** | 0.682 | 0.875 | **0.031** | 0.246 | 0.531 | 0.714 | 0.216 | 0.904 | 1.000 | 0.328 | 0.162 | 0.402 | 0.930 | 0.727 | 0.846 | 0.071 | 0.677 |
|  |  |  |  |  |  |  |  |  |  |  |  |  |  |  |  |  |  |  |
| **TbD** | **N** | 45 | 45 | 45 | 45 | 45 | 45 | 45 | 45 | 45 | 45 | 45 | 45 | 44 | 44 | 45 | 44 | 45 |
|  | **NA** | 7.0 | 6.2 | 9.3 | 7.5 | 5.6 | 3.3 | 7.2 | 3.0 | 3.3 | 4.3 | 2.0 | 6.6 | 18.3 | 10.8 | 3.9 | 9.4 | 12.3 |
|  | **NAP** | 0.0 | 0.0 | 0.0 | 0.2 | 0.4 | 0.1 | 0.3 | 0.0 | 0.0 | 0.0 | 0.0 | 0.0 | 0.8 | 0.1 | 0.0 | 0.0 | 0.4 |
|  | **Ho** | 0.800 | 0.733 | 0.622 | 0.667 | 0.422 | 0.333 | 0.711 | 0.444 | 0.089 | 0.444 | 0.400 | 0.822 | 0.864 | 0.841 | 0.489 | 0.864 | 0.844 |
|  | **He** | 0.753 | 0.718 | 0.691 | 0.760 | 0.408 | 0.400 | 0.767 | 0.437 | 0.107 | 0.489 | 0.369 | 0.777 | 0.919 | 0.829 | 0.612 | 0.838 | 0.874 |
|  | **H-W-test** | 0.729 | 0.375 | 0.512 | 0.212 | 0.618 | 0.581 | 0.068 | 1.000 | 0.184 | 0.577 | 1.000 | 0.700 | 0.157 | 0.706 | 0.245 | 0.410 | 0.734 |
|  |  |  |  |  |  |  |  |  |  |  |  |  |  |  |  |  |  |  |
| **TbS** | **N** | 25 | 25 | 25 | 25 | 25 | 25 | 25 | 25 | 25 | 25 | 25 | 25 | 24 | 24 | 25 | 25 | 25 |
|  | **NA** | 7.0 | 8.0 | 11.0 | 4.0 | 4.0 | 3.0 | 7.0 | 3.0 | 4.0 | 5.0 | 2.0 | 7.0 | 15.0 | 11.0 | 4.0 | 9.0 | 11.0 |
|  | **NAP** | 0.1 | 0.2 | 0.1 | 0.0 | 1.0 | 0.0 | 0.0 | 0.0 | 0.0 | 0.0 | 0.0 | 0.2 | 0.0 | 0.0 | 0.0 | 0.1 | 0.6 |
|  | **Ho** | 0.760 | 0.800 | 0.840 | 0.760 | 0.280 | 0.240 | 0.600 | 0.520 | 0.240 | 0.480 | 0.200 | 0.760 | 0.875 | 0.833 | 0.320 | 0.640 | 0.880 |
|  | **He** | 0.660 | 0.776 | 0.820 | 0.769 | 0.286 | 0.284 | 0.776 | 0.593 | 0.221 | 0.484 | 0.180 | 0.769 | 0.818 | 0.804 | 0.403 | 0.804 | 0.877 |
|  | **H-W-test** | 0.063 | 0.815 | 0.833 | 0.396 | 1.000 | 0.125 | 0.119 | 0.761 | 1.000 | 0.236 | 1.000 | 0.134 | 0.698 | 0.499 | 0.308 | **0.030** | 0.632 |
|  |  |  |  |  |  |  |  |  |  |  |  |  |  |  |  |  |  |  |
| **SbL** | **N** | 62 | 62 | 62 | 62 | 62 | 62 | 62 | 62 | 62 | 61 | 61 | 61 | 61 | 62 | 62 | 62 | 62 |
|  | **NA** | 7.2 | 6.4 | 11.5 | 7.1 | 5.3 | 3.0 | 6.7 | 3.0 | 3.3 | 4.9 | 2.0 | 6.4 | 15.7 | 11.1 | 4.0 | 9.5 | 13.3 |
|  | **NAP** | 0.0 | 0.0 | 0.5 | 0.0 | 0.4 | 0.0 | 0.0 | 0.0 | 0.0 | 0.0 | 0.0 | 0.4 | 0.9 | 0.0 | 0.0 | 0.3 | 0.2 |
|  | **Ho** | 0.726 | 0.694 | 0.839 | 0.742 | 0.323 | 0.097 | 0.565 | 0.629 | 0.161 | 0.525 | 0.180 | 0.705 | 0.934 | 0.952 | 0.516 | 0.774 | 0.855 |
|  | **He** | 0.776 | 0.756 | 0.853 | 0.768 | 0.340 | 0.123 | 0.722 | 0.576 | 0.166 | 0.487 | 0.216 | 0.746 | 0.908 | 0.828 | 0.565 | 0.838 | 0.903 |
|  | **H-W-test** | 0.612 | 0.852 | 0.934 | 0.424 | 0.392 | 0.209 | **0.004** | 0.917 | 0.387 | 0.202 | 0.207 | **0.048** | 0.254 | 0.531 | 0.493 | 0.139 | 0.356 |
|  |  |  |  |  |  |  |  |  |  |  |  |  |  |  |  |  |  |  |
| **SbD** | **N** | 41 | 41 | 41 | 41 | 41 | 41 | 41 | 41 | 41 | 41 | 41 | 41 | 41 | 41 | 41 | 41 | 41 |
|  | **NA** | 6.8 | 7.0 | 8.2 | 8.3 | 5.8 | 3.5 | 6.9 | 3.0 | 4.8 | 4.9 | 2.0 | 6.0 | 14.4 | 11.7 | 3.9 | 8.7 | 13.6 |
|  | **NAP** | 0.0 | 0.6 | 0.0 | 0.0 | 0.1 | 0.2 | 0.0 | 0.0 | 1.0 | 0.0 | 0.0 | 0.0 | 0.5 | 0.0 | 0.0 | 0.1 | 0.1 |
|  | **Ho** | 0.756 | 0.805 | 0.659 | 0.732 | 0.366 | 0.293 | 0.683 | 0.415 | 0.293 | 0.732 | 0.439 | 0.756 | 0.805 | 0.829 | 0.585 | 0.780 | 0.854 |
|  | **He** | 0.778 | 0.760 | 0.749 | 0.779 | 0.439 | 0.276 | 0.736 | 0.511 | 0.283 | 0.632 | 0.450 | 0.750 | 0.859 | 0.836 | 0.620 | 0.813 | 0.883 |
|  | **H-W-test** | 0.462 | 0.384 | 0.130 | 0.621 | 0.080 | 0.195 | 0.297 | 0.106 | 0.440 | 0.578 | 1.000 | 0.940 | 0.396 | 0.451 | 0.231 | 0.335 | **0.040** |
|  |  |  |  |  |  |  |  |  |  |  |  |  |  |  |  |  |  |  |
| **SbS** | **N** | 61 | 61 | 61 | 61 | 61 | 61 | 61 | 61 | 61 | 61 | 61 | 61 | 60 | 60 | 60 | 61 | 61 |
|  | **NA** | 6.4 | 6.7 | 9.2 | 7.1 | 4.7 | 3.8 | 6.4 | 3.0 | 2.6 | 4.0 | 2.7 | 7.5 | 14.1 | 10.5 | 4.5 | 9.0 | 12.6 |
|  | **NAP** | 0.0 | 0.3 | 0.0 | 0.0 | 0.6 | 0.8 | 0.0 | 0.0 | 0.0 | 0.0 | 0.7 | 0.0 | 0.5 | 0.0 | 0.5 | 0.0 | 0.0 |
|  | **Ho** | 0.541 | 0.770 | 0.721 | 0.656 | 0.213 | 0.180 | 0.607 | 0.590 | 0.082 | 0.393 | 0.311 | 0.770 | 0.883 | 0.883 | 0.350 | 0.820 | 0.836 |
|  | **He** | 0.556 | 0.786 | 0.802 | 0.786 | 0.199 | 0.184 | 0.719 | 0.599 | 0.080 | 0.420 | 0.290 | 0.769 | 0.895 | 0.844 | 0.334 | 0.789 | 0.900 |
|  | **H-W-test** | 0.240 | 0.397 | 0.448 | **0.016** | 1.000 | 0.258 | 0.308 | 1.000 | 1.000 | 0.345 | 1.000 | 0.165 | 0.121 | 0.859 | 0.761 | 0.671 | 0.354 |
|  |  |  |  |  |  |  |  |  |  |  |  |  |  |  |  |  |  |  |
| **LFL** | **N** | 65 | 69 | 69 | 69 | 69 | 69 | 69 | 69 | 69 | 69 | 69 | 69 | 69 | 69 | 69 | 69 | 67 |
|  | **NA** | 6.9 | 6.6 | 10.2 | 8.0 | 6.0 | 2.7 | 7.0 | 3.0 | 3.8 | 5.8 | 2.0 | 7.0 | 15.2 | 11.1 | 4.7 | 8.8 | 10.9 |
|  | **NAP** | 0.0 | 0.2 | 0.2 | 0.0 | 0.4 | 0.0 | 0.2 | 0.0 | 0.5 | 0.1 | 0.0 | 0.3 | 0.6 | 0.1 | 0.1 | 0.0 | 0.0 |
|  | **Ho** | 0.846 | 0.783 | 0.855 | 0.725 | 0.449 | 0.188 | 0.725 | 0.710 | 0.246 | 0.594 | 0.391 | 0.841 | 0.942 | 0.797 | 0.536 | 0.826 | 0.881 |
|  | **He** | 0.826 | 0.778 | 0.822 | 0.741 | 0.439 | 0.186 | 0.757 | 0.589 | 0.224 | 0.600 | 0.379 | 0.755 | 0.874 | 0.785 | 0.589 | 0.785 | 0.838 |
|  | **H-W-test** | 0.834 | 0.611 | 0.956 | 0.445 | 0.695 | 0.283 | 0.716 | **0.019** | 1.000 | 0.707 | 1.000 | 0.988 | 0.694 | 0.974 | 0.549 | 0.741 | **0.031** |
|  |  |  |  |  |  |  |  |  |  |  |  |  |  |  |  |  |  |  |
| **LFD** | **N** | 65 | 65 | 65 | 65 | 65 | 65 | 65 | 64 | 65 | 65 | 65 | 65 | 65 | 65 | 65 | 65 | 62 |
|  | **NA** | 6.6 | 5.9 | 6.2 | 8.1 | 5.1 | 2.4 | 6.9 | 3.0 | 3.6 | 4.8 | 2.0 | 7.3 | 14.7 | 11.9 | 4.3 | 9.4 | 13.1 |
|  | **NAP** | 0.0 | 0.0 | 0.0 | 0.0 | 0.1 | 0.0 | 0.0 | 0.0 | 0.0 | 0.0 | 0.0 | 0.5 | 0.2 | 0.0 | 0.4 | 0.6 | 0.1 |
|  | **Ho** | 0.785 | 0.738 | 0.615 | 0.738 | 0.323 | 0.185 | 0.738 | 0.578 | 0.215 | 0.538 | 0.446 | 0.769 | 0.785 | 0.831 | 0.600 | 0.785 | 0.887 |
|  | **He** | 0.782 | 0.754 | 0.705 | 0.723 | 0.346 | 0.169 | 0.808 | 0.538 | 0.211 | 0.557 | 0.462 | 0.731 | 0.821 | 0.811 | 0.667 | 0.835 | 0.869 |
|  | **H-W-test** | 0.813 | 0.482 | 0.564 | 0.432 | 0.265 | 1.000 | 0.207 | 0.781 | 0.427 | 0.694 | 0.792 | 0.166 | 0.220 | 0.939 | 0.463 | 0.084 | 0.914 |
|  |  |  |  |  |  |  |  |  |  |  |  |  |  |  |  |  |  |  |
| **LFS** | **N** | 55 | 55 | 55 | 55 | 55 | 55 | 55 | 55 | 55 | 55 | 55 | 55 | 55 | 55 | 55 | 55 | 55 |
|  | **NA** | 6.9 | 5.9 | 9.6 | 8.2 | 6.4 | 3.0 | 6.3 | 3.0 | 3.1 | 5.3 | 2.0 | 7.1 | 13.0 | 8.2 | 5.0 | 8.2 | 10.2 |
|  | **NAP** | 0.0 | 0.0 | 0.1 | 0.4 | 0.5 | 0.0 | 0.0 | 0.0 | 0.0 | 0.1 | 0.0 | 0.0 | 0.3 | 0.1 | 0.6 | 0.0 | 0.0 |
|  | **Ho** | 0.836 | 0.709 | 0.764 | 0.673 | 0.509 | 0.255 | 0.691 | 0.545 | 0.145 | 0.564 | 0.291 | 0.836 | 0.909 | 0.691 | 0.691 | 0.745 | 0.782 |
|  | **He** | 0.807 | 0.699 | 0.799 | 0.737 | 0.560 | 0.259 | 0.700 | 0.609 | 0.138 | 0.538 | 0.249 | 0.745 | 0.895 | 0.733 | 0.676 | 0.726 | 0.845 |
|  | **H-W-test** | 0.844 | 0.777 | 0.536 | 0.456 | 0.408 | 0.213 | 0.857 | **0.016** | 1.000 | 0.603 | 0.581 | 0.645 | 0.691 | 0.078 | 0.572 | 0.732 | **0.013** |

Fig S1. Probability of the number of clusters present in the data using a hierarchical approach on the full dataset (A), and the subsequent 2^nd^ (B, C) and 3^rd^ (D) rounds.


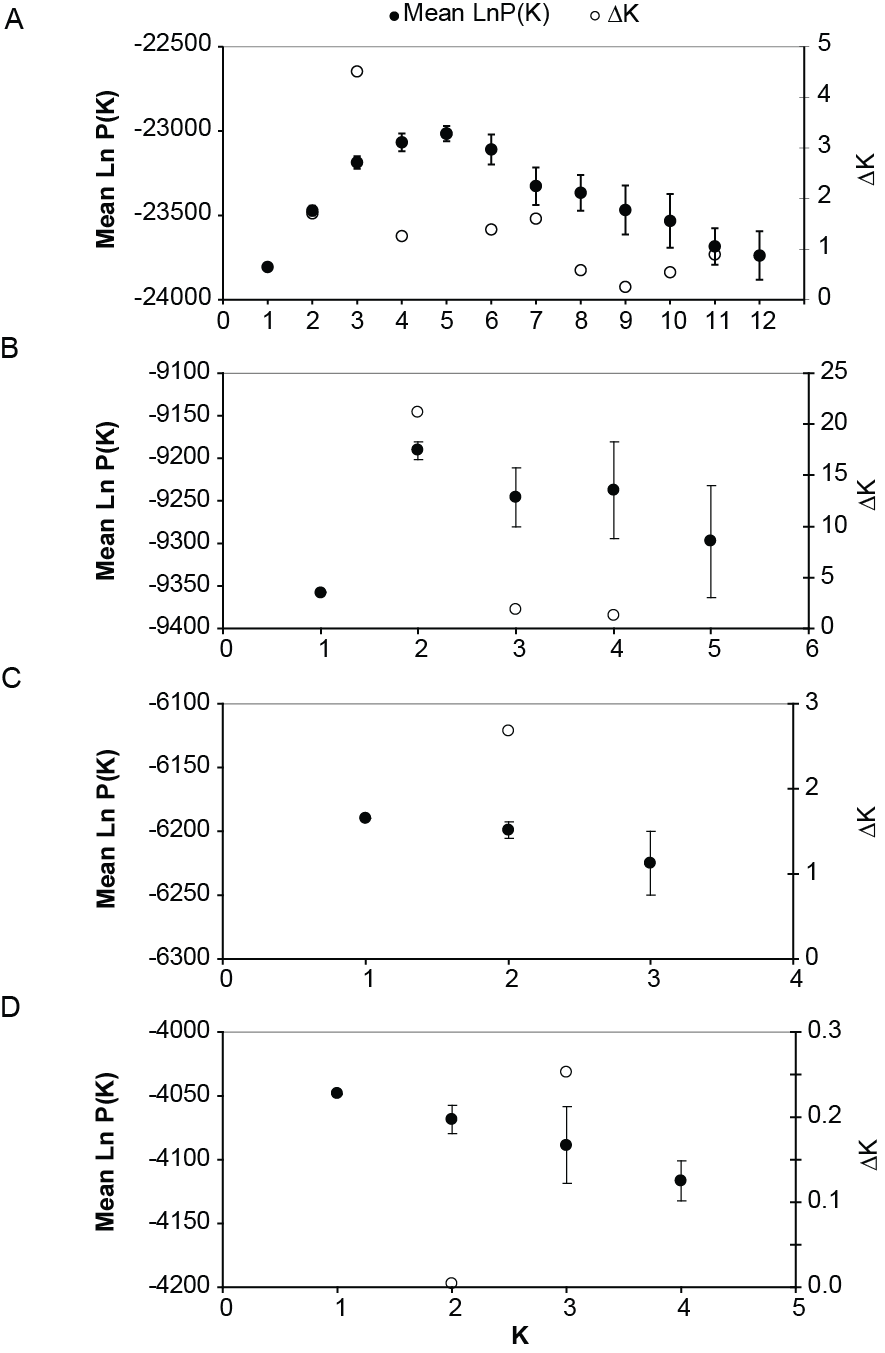


**References**

Præbel K, Westgaard J-I, Amundsen P-A*, et al.* (2013) A diagnostic tool for efficient analysis of population structure, hybridization and conservation status of European whitefish (*Coregonus lavaretus* (L.)) and vendace (*C. albula* (L.)). *Fundamental and Applied Limnology - Advances in Limnology* **64**, 247-255.

Rice WR (1989) Analyzing tables of statistical tests. *Evolution* **43**, 223-225.
